# Working Memory Load Effects on the Tilt Aftereffect

**DOI:** 10.3389/fpsyg.2021.618712

**Published:** 2021-06-15

**Authors:** Gaoxing Mei, Mofen Cen, Xu Luo, Shiming Qiu, Yun Pan

**Affiliations:** Department of Psychology, School of Psychology, Guizhou Normal University, Guiyang, China

**Keywords:** attention, tilt aftereffect, visual adaptation, visual load, working memory

## Abstract

Prolonged exposure to an oriented stimulus causes a subsequent test stimulus to be perceived as tilted in the opposite direction, a phenomenon referred to as the tilt aftereffect (TAE). Previous studies have demonstrated that high-level cognitive functions such as attention can modulate the TAE, which is generally well-known as a low-level perceptual process. However, it is unclear whether working memory load, another high-level cognitive function, could modulate the TAE. To address this issue, here we developed a new paradigm by combining a working memory load task with a TAE task. Participants firstly remembered a stream of digits (Experiment 1) or four color-shape conjunctions (Experiment 2) under high/low load conditions, and then recognized the probe stimuli (digits or a color-shape conjunction), which were presented at the center of an adapting grating. After the recognition task (i.e., the adaptation stage), participants performed an orientation judgment task to measure their TAEs. The result of Experiment 1, where the load stimuli were digits, showed that the magnitude of the TAEs were reduced under the condition of the high working memory load compared to that of the low working memory load. However, we failed to replicate the finding in Experiment 2, where the load stimuli were color-shape conjunctions. Together, our two experiments provided mixed evidence regarding the working memory load effects on the TAE and further replications are needed in future work.

## Introduction

Maintaining high sensitivity to an ever-changing visual environment is a fundamental characteristic of human visual system. This function is primarily manifested by visual adaptation, which refers to a perceptual phenomenon in which prolonged viewing of a visual stimulus can produce a significant visual negative aftereffect (for reviews, Clifford et al., 2007; Kohn, 2007; Webster, 2011, 2015). A typical example is the tilt aftereffect (TAE), in which prolonged exposure to an oriented stimulus (e.g., a grating tilted at 15° clockwise from vertical) causes a subsequent vertical stimulus (e.g., a vertical grating) to appear to be tilted in the opposite direction (e.g., slightly counterclockwise tilt from vertical) (Gibson and Radner, 1937).

Although it is well-known that the TAE is a low-level perceptual process in the primary visual cortex (e.g., Blakemore et al., 1970), several psychophysical results have shown that attention, as a high-level cognitive function, can increase the magnitude of the TAE (Spivey and Spirn, 2000; Festman and Ahissar, 2004; Montaser-Kouhsari and Rajimehr, 2004; Jung and Chong, 2014; Pavan et al., 2016). For example, in the study of Spivey and Spirn (2000), participants were instructed to selectively attend to one of the two oriented gratings during adaptation while maintaining fixation on a central dot, and then the TAEs were measured for the attended and the unattended position. The results suggested a larger magnitude of the TAE for the attended position compared to that for the unattended position. Furthermore, a neuroimaging study suggested that a lower fMRI response in visual cortex was found for the attended orientation compared to the unattended orientation, and moreover, the magnitude of the psychophysical TAE had a significant correlation with that of the fMRI response adaptation in V1 (Liu et al., 2007). These psychophysical and neuroimaging results indicate that attention modulates the low-level TAE originated from the primary visual cortex.

As another high-level cognitive function, working memory is closely associated with attention (for a recent review, Oberauer, 2019). Working memory and attention share cognitive resources (e.g., Cowan et al., 2005; Chen and Cowan, 2009; Morey and Bieler, 2012). Previous studies have shown that high working memory load can reduce the amount of available attention resources (e.g., Lavie et al., 2004; Pratt et al., 2011; Wei and Zhou, 2020), and working memory load can also influence visual processing such as visual search (Konstantinou and Lavie, 2013; Berggren and Eimer, 2018), scene-viewing (Cronin et al., 2020), and even contrast detection (Liu et al., 2018). However, whether and how working memory load can modulate visual aftereffects remain largely unknown.

The role of working memory on visual aftereffects can be investigated from two aspects. One aspect is how working memory contents would affect visual aftereffects (Kang et al., 2011; Saad and Silvanto, 2013). For example, in Saad and Silvanto (2013) an enhanced TAE was found when the orientation of memory contents (i.e., gratings) was congruent relative to incongruent with that of the adapting contents (i.e., adaptors). Another aspect is to what extent working memory load would modulate visual aftereffects when memory contents have no relations with the adapting stimuli. To our knowledge, no study investigated how different levels of working memory load would affect visual aftereffects. Some previous studies compared the magnitude of visual aftereffects under a memory load condition with that under a passive viewing condition (Moradi et al., 2005; Blaser and Shepard, 2009). For example, the magnitude of the face identity aftereffect was reduced when participants performed a visual working memory task with high-load relative to passive viewing (Moradi et al., 2005). A recent study has revealed that working memory load also modulates early visual perceptual processing (Liu et al., 2018). Thus, we hypothesized that high relative to low working memory load would reduce low-level visual aftereffects such as the TAE.

Here, we aimed to examine whether different levels of visual working memory load (high or low) could modulate the TAE, by combining a digit memory task (Experiment 1) or a color-shape conjunction memory task (Experiment 2) with the TAE task. Our results showed that high relative to low working memory load reduced the magnitude of the TAE when the digit memory task was used; however, this finding failed to be replicated when the color-shape conjunction memory task was used.

## Experiment 1

### Methods

#### Participants

Seventeen naïve volunteers (15 females; *M* = 20.5 years, *SD* = 2.3; age range, 17–25 years) participated in Experiment 1. One additional participant was excluded because of her very low correct rate for detecting the letter X in the no-memory load condition (see section Procedure for details). All participants in the present study had normal or corrected-to-normal vision, and provided written informed consent for participation. The study was approved by the School of Psychology Ethics Committee at Guizhou Normal University, and conformed to the Declaration of Helsinki (World Medical Association, 2013).

#### Stimuli and Apparatus

Adapting stimuli and test stimuli were sinusoidal gratings with a spatial frequency of 1.44 cpd and a diameter of 6°. Their edges were smoothed in a Gaussian profile. The Michelson contrasts of adapters and TAE probes were 0.9 and 0.5, respectively. The orientation of the adapting stimuli was +15° clockwise from vertical or −15° counterclockwise from vertical; the test stimuli included 21 tilt orientations ranging from −5° to +5° with a 0.5° increment from vertical. In addition, Arabic digits (1–9) were used for the working memory load task. A single digit subtended a visual angle of 0.4° × 0.4°. All experiments were programmed using MATLAB software (MathWorks, Natick, MA) and PsychToolbox-3 (Brainard, 1997; Pelli, 1997). The stimuli were displayed on the center of a gamma-corrected Philips 201P10 CRT monitor (21-in; resolution, 1,024 × 768 pixels; refresh rate, 85 Hz). Participants viewed stimuli at a distance of 57 cm, with the help of a chinrest.

#### Procedure

**Figure 1:**
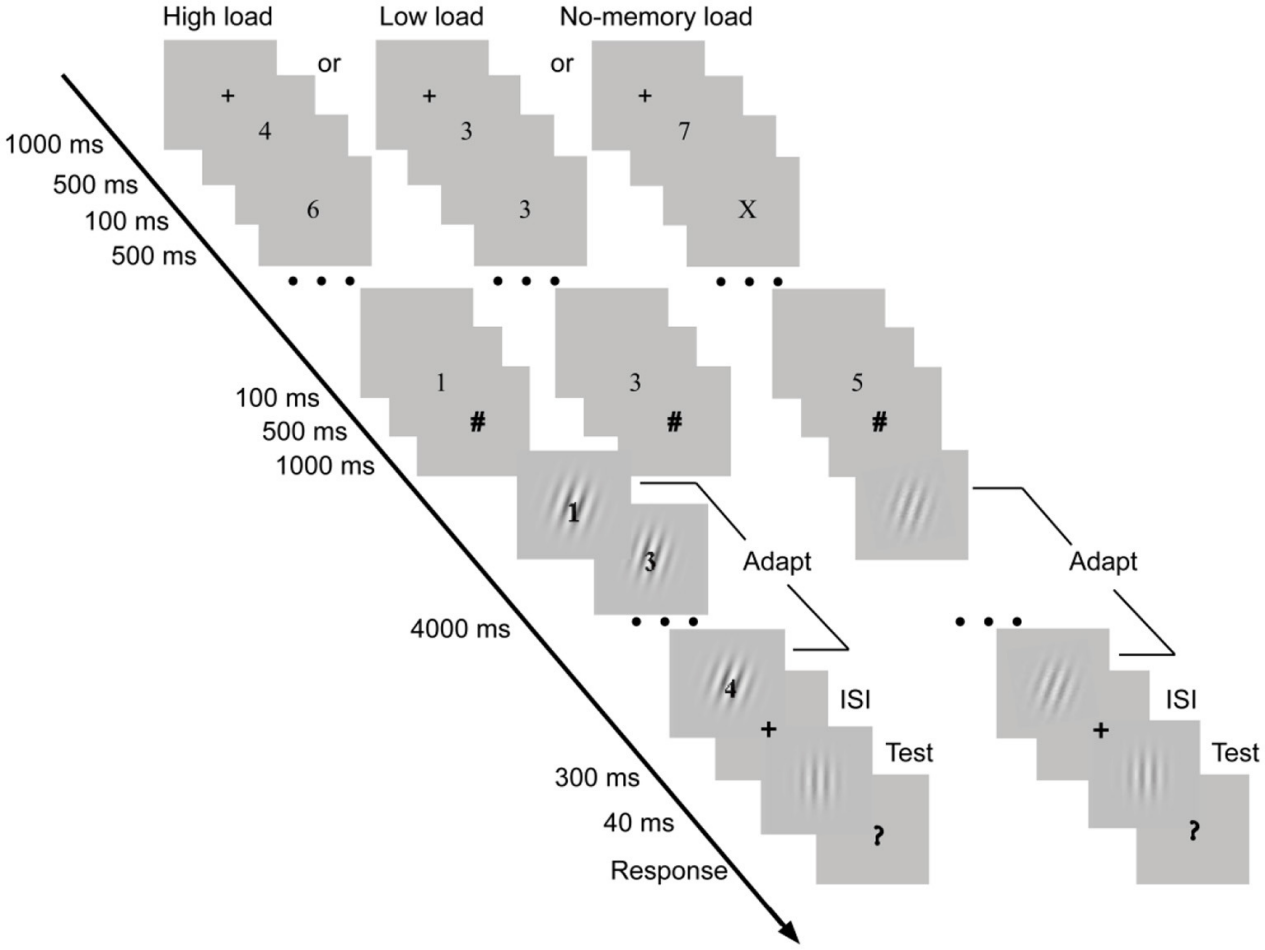
Trial procedures of the low-load, high-load, and no-memory load condition in Experiment 1. For the low-load and high-load condition, in each trial participants performed two tasks of the working memory load and the tilt aftereffect (TAE). After a 1,000-ms fixation cross (+), six unduplicated digits (the high-load condition) or identical digits (the low-load condition) were consecutively presented on the center of screen, each lasting for 500 ms. Participants were required to remember these digits. Then a stream of probe digits appeared on top of an adapting grating, and participants decided whether the probe digits had appeared during memory stage. After a 300-ms inter-stimulus interval (ISI), the test stimulus appeared for 40 ms, and then participants were instructed to decide that the test grating was perceived as either clockwise or counter-clockwise tilt from vertical. For the no-memory load condition, the trial procedure was similar to that under the high-load condition except the followings. Instead of the digit memory task under the high-load condition, a rapid serial visual presentation task (RSVP) was conducted (See this figure). Participants were instructed to press the space button when the letter X appeared in the RSVP sequence. The letter X could appear once or twice in each trial. In addition, during the adaptation stage the digit recognition task was also removed and participants just passively viewed the adapting stimulus.

There were three experimental conditions: a high-load, a low-load, and a no-memory load condition. Another three corresponding no-adaptation control conditions were also used. For the high- and low-load conditions, we followed the experimental paradigm of Zäske et al. (2016). In each trial of the high-load condition, six unduplicated digits were consecutively presented after a 1,000-ms fixation cross (see Figure 1). Each digit was randomly selected among 1–9, and was presented for 500 ms at the center of the screen. The time interval between two digits was 100 ms. Participants were required to remember the six digits. After the presentation of the last digit, a prompt sign (#) was presented for 1,000 ms, indicating a forthcoming recognition test. Then a probe digit appeared on top of an adapting grating whose orientation (+15° or−15°) was counterbalanced across participants. Participants were instructed to press the “Y” key as quickly and accurately as possible with their left hand if the probe digit had just been remembered but to press the “N” key if the probe digit had not been remembered. The probe digit was also randomly selected among 1–9. Once participants made a response, a next probe digit again appeared on the adapting grating. Participants were required to continue to perform the digit recognition task until the adapting grating, which was presented for 4,000 ms, disappeared. After a 300-ms blank interval, a test grating was presented for 40 ms. The orientation of the test grating was selected from the 21 tilt orientations according to a one-down-one-up staircase procedure (Levitt, 1971). For example, if participants perceived the orientation of the test grating as clockwise/counter-clockwise tilt in the current trial, in the next trial the test grating would be presented with counter-clockwise/clockwise tilt of 0.5° relative to the orientation of the test grating of the previous trial. The initial orientation of the test grating in each block was 0° (i.e., a vertical grating). When a question mark (“?”) appeared, participants were instructed to press the right/left arrow with their right hand if they perceived the test grating as clockwise/counter-clockwise tilt from vertical. The next trial began until participants responded. For the low-load condition, a similar trial procedure was conducted, except that during the digit memory stage only an identical digit (e.g., 3) was presented six times, each lasting for 500 ms. A 100-ms time interval was used between two times (see Figure 1). Given that more digits were required to be remembered in the high-load condition than that in the low-load condition, we hypothesized that more high memory load was involved in the high-load condition. We would examine whether this manipulation was successful by comparing the correct reaction rates and reaction times for these two conditions during the digit recognition stage. For the no-memory load condition, the trial procedure was similar to that under the high-load condition except the followings. A rapid serial visual presentation task (RSVP), instead of the digit memory task under the high-load condition, was conducted (See Figure 1). Participants decided whether the letter X appeared in the RSVP sequence by pressing the space button. The letter X could appear once or twice in each trial. In addition, during the adaptation stage the digit recognition task was also removed and participants just passively viewed the adapting stimulus. We designed the no-load condition to measure the TAEs without any interference from the working memory load task, and thus it was possible to examine whether the working memory load would increase or decrease the magnitude of the TAE. We expected a largest TAE under the no-memory load condition. The procedures of the three no-adaptation control conditions were the same as those used in the corresponding experimental conditions (i.e., the high-load, low-load, and no-memory load condition), except that the adapting stimuli were vertical gratings. The vertical adaptors were used in the no-adaptation control conditions because they are not expected to induce any systematic orientation change on vertical test stimuli (e.g., Gibson, 1933; Campbell and Maffei, 1971; Held and Shattuck, 1971; Vaitkevicius et al., 2009; Pavan et al., 2016).

Each condition was performed in separate blocks and was repeated four times. Thus, each participant performed 24 blocks, and each block included 48 trials. Participants performed practices before the formal test. Each block took approximately 10 min, and thus approximately 4 h in total were required for 24 blocks. Participants had a break for at least 30 min between two successive blocks and performed all blocks across several days.

#### Analysis

In each block the average tilt of the last 10 reversals was used as the magnitude of the TAE for the corresponding block. For each participant, the mean of four blocks for each condition was used as the magnitude of the TAE of the corresponding condition. To remove individuals' bias in orientation judgments, then the TAEs for statistical tests were calculated by subtracting the magnitude of the TAE in the corresponding no-adaptation control condition from the magnitude in the adaptation condition (i.e., the high-load, low-load, and no-memory load condition). Given that participants needed to perform the digit recognition task during adaptation stage, the grating adaptors should be away from their attentional focus. If adaptation failed to occur, the magnitude of the TAE would be zero for each experimental condition. Thus, to examine whether the TAE was significant in each condition, we used one-sample *t*-test to compare the magnitude of the TAE with zero. The Cohen's *d* was used as the estimate of the effect size for the *t*-tests (Cohen, 1992). The One-Way Repeated Measures ANOVA was used to compare the differences of the TAEs under the high-load, low-load and no-memory load condition. We used partial eta-squared ($\eta_{\text{p}}^{2}$) to evaluate the effect size for the ANOVA (Cohen, 1973).

### Results

To examine whether the manipulation of the working memory load was successful, we compared the correct reaction rates and reaction times under the low-load condition with those under the high-load condition (Zäske et al., 2016). The results of paired *t*-tests showed higher correct reaction rates and faster reaction times under the low load condition than under the high load condition [96.4 vs. 87.1%, *t*_(16)_ = 3.72, *p* = 0.002, *d* = 0.93; 601 vs. 822 ms, *t*_(16)_ = 15.44, *p* < 0.001, *d* = 3.86], indicating a successful manipulation of the working memory load.

**Figure 2:**
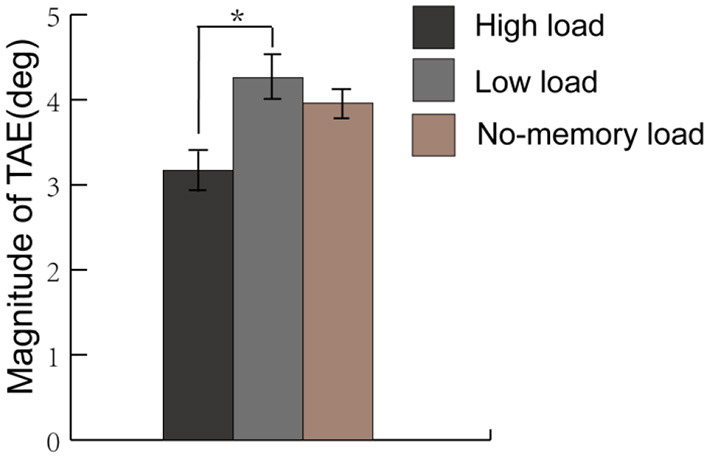
The average magnitude of the tilt aftereffect (TAE) under the low-load, high-load, and no-memory load condition in Experiment 1. High relative to low working memory load reduced the magnitude of the TAEs. The error bars show ±1 SEM. *p < 0.05.

After removing individuals' bias by subtracting the magnitudes in the no-adaptation conditions, one-sample *t*-tests showed that significant TAEs were also found in all three conditions [see Figure 2; high-load condition: 3.17°, *t*_(16)_ = 5.69, *p* < 0.001, *d* = 1.42; low-load condition: 4.26°, *t*_(16)_ = 7.44, *p* < 0.001, *d* = 1.42; no-memory condition: 3.96°, *t*_(16)_ = 5.80, *p* < 0.001, *d* = 1.45]. These results indicated that the TAE still appeared despite being away from attentional focus for the grating adaptors. Unexpectedly, we failed to obtain the largest magnitude of the TAE under the no-memory condition.

The One-Way Repeated Measures ANOVA showed that there was a marginal significant main effect of the condition on the magnitude of TAE [*F*_(2, 32)_ = 3.014, *p* = 0.063, $\eta_{\text{p}}^{2}$ = 0.159]. Then *Post-hoc* pairwise comparisons among the high-load, low-load, and no-load conditions were performed with Bonferonni correction. Importantly, the results demonstrated a stronger magnitude of TAEs under the low relative to high working load condition (4.26° vs. 3.17°, *p* = 0.010), indicating that high relative to low working memory load attenuated the magnitude of the TAE. No significant differences were found for other paired comparisons.

## Experiment 2

The purpose of Experiment 2 was simply to perform a replication using a different stimulus of memory load (i.e., the color-shape conjunction), in order to examine the generality of working memory load effects on the TAE. The color-shape conjunction has widely been used as the stimulus of memory load in previous studies (e.g., Lavie, 1995; Luck and Vogel, 1997; Qian et al., 2019).

### Methods

#### Participants

Seventeen naïve volunteers (16 females; *M* = 22.5 years, *SD* = 2.5; age range, 18–26 years) participated in Experiment 2. All participants had no color blindness. One additional participant was excluded because of her very low correct rate in the color detection task for the no-memory load condition (see section Procedure in details).

#### Stimuli, Apparatus, Procedure, and Analysis

**Figure 3:**
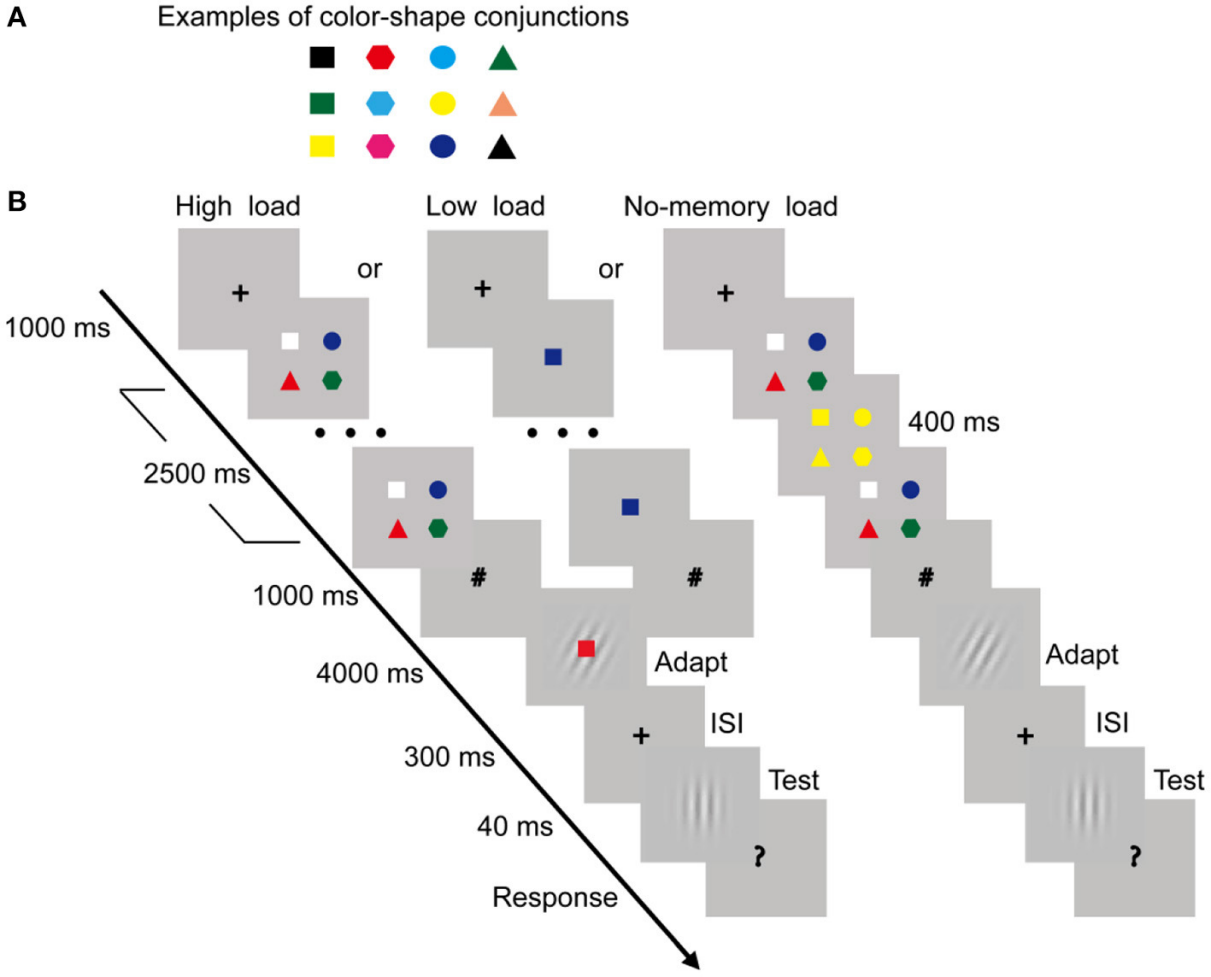
Examples of color-shape conjunctions **(A)** and trial procedures of the low-load, high-load, and no-memory load condition **(B)** in Experiment 2. The same trial procedure as that of Experiment 1 was used, except that the color-shape conjunctions rather than digits were used in the working memory load task (see section Procedure for details).

The stimuli, procedure, and analysis for the TAE task were same as those in Experiment 1 except the followings. Instead of digits in Experiment 1, the color-shape conjunction (see examples in Figure 3A, visual angle, 0.42° × 0.42°) was used as the stimulus for the working memory load task. Each conjunction consisted of two features: color and shape. The optional colors included nine categories: black (RGB: 0 0 0), white (RGB: 255 255 255), red (RGB: 255 0 0), yellow (RGB: 255 255 0), green (RGB: 0 255 0), cyan (RGB: 0 255 255), blue (RGB: 0 0 255), pink (RGB: 255 127 80), and magenta (RGB: 255 0 255); the optional shapes included four categories: square, circle, triangle, and hexagon. The same apparatus as Experiment 1 were used.

Like Experiment 1, the three experimental conditions (i.e., the high-load, low-load, and no-memory load) and the three corresponding no-adaptation control conditions were included. For the working memory load task, four color-shape conjunctions and a color-shape conjunction were used for the high-load condition and the low-load condition, respectively. In each trial of the high-load condition (see Figure 3B), after a 1,000-ms fixation cross, four color-shape conjunctions centered 2.4° away from the fixation were presented for 2,500 ms on the diagonal of four quadrants. To reduce the difficulty of the task, each shape was presented at a fixed position in all trials. Specifically, square, circle, triangle, and hexagon were always presented at the upper-left, upper-right, bottom-left and bottom-right position, respectively. The color of each conjunction was randomly selected from the nine categories; the colors of the conjunctions at four positions were different from each other in each trial. Participants were required to remember the four color-shape conjunctions (i.e., both color and shape needed be remembered for each conjunction). After a 1,000-ms prompt sign (#), the similar TAE task as Experiment 1 began. In order to reduce the difficulty of the task according to our pilot test, only a color-shape conjunction (i.e., the probe conjunction) was presented on top of the adapting grating. Participants were instructed to press the “Y” key with their left hand if the probe conjunction was one of four remembered color-shape conjunctions; otherwise, press the “N” key. In half of the trials, the probe conjunction had appeared in the working memory load task; in the other half, the probe conjunction had not appeared. The probe conjunction and the adapting grating were simultaneously presented. Once participants made a response, the probe conjunction would disappear. The measurement of the TAE was completely same as Experiment 1. For the no-memory load condition (see Figure 3), a similar trial procedure as that of the high load condition was used, except that a color detection task rather than the memory task was performed, and the recognition task was removed during adaptation stage (i.e., only the adapting stimulus was presented). In the color detection task, participants were instructed to press the space button when the colors of the four color-shape conjunctions were changed into a randomly uniform color. The change appeared between 1 and 2 s in the 2.5-s duration and lasted for 400 ms. Like Experiment 1, in the three no-adaptation control conditions vertical gratings were used. In addition, the same analysis method and the number of blocks as those in Experiment 1 were used.

### Results

**Figure 4:**
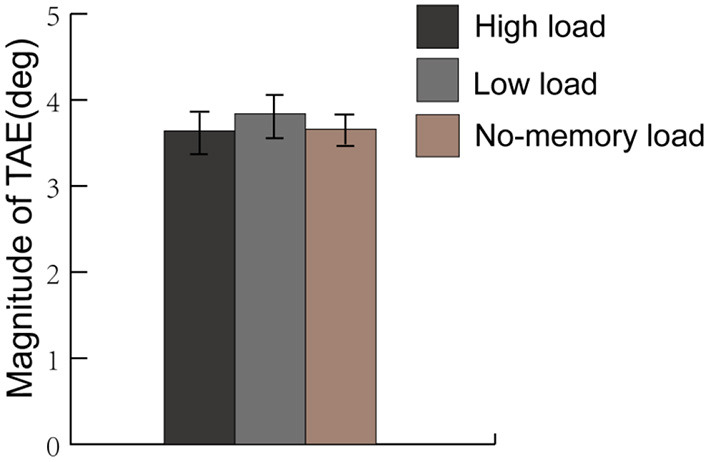
The average magnitude of the TAEs under the low-load, high-load, and no-memory load condition in Experiment 2. No significant main effects between all three conditions on the magnitude of TAE was found.

Consistently with the results of Experiment 1, higher correct reaction rates and faster reaction times under the low load condition in the color-shape conjunction recognition task were found when compared to those under the high load condition [97.3 vs. 82.1%, *t*_(16)_ = 9.04, *p* < 0.001, *d* = 2.26; 767 vs. 1,230 ms, *t*_(16)_ = 11.68, *p* < 0.001, *d* = 2.92], indicating a successful manipulation regarding the working memory load. Significant TAEs were still found in all three experimental conditions when the strengths of the TAEs were removed from the three corresponding control conditions [see Figure 4; the high-load condition: 3.64°, *t*_(16)_ = 8.53, *p* < 0.001, *d* = 2.13; the low-load condition: 3.84°, *t*_(16)_ = 9.51, *p* < 0.001, *d* = 2.38; the no-memory condition: 3.66°, *t*_(16)_ = 9.42, *p* < 0.001, *d* = 2.36]. However, inconsistent with the results of Experiment 1, the One-Way Repeated Measures ANOVA showed no significant main effect of the condition on the magnitude of TAE [*F*_(2, 32)_ = 0.399, *p* = 0.565, $\eta_{\text{p}}^{2}$ = 0.024]. Taken together, the results of Experiment 1 and 2 demonstrated that the modulation of working memory load on the magnitude of TAE depended on load tasks.

## Discussion

By combining the working memory load task and the TAE task, we found that the magnitudes of the TAEs were reduced under the high relative to low load condition when the digit load stimuli were used in Experiment 1. However, there was no significant difference between the high and low load condition when the color-shape conjunctions were used in Experiment 2. In other words, Experiment 2 failed to replicate the findings of Experiment 1. The current study provided mixed evidence regarding the working memory load effects on the TAE.

The results of Experiment 1 are consistent with previous studies regarding the modulating effect of attention toward the TAE (Spivey and Spirn, 2000; Festman and Ahissar, 2004; Montaser-Kouhsari and Rajimehr, 2004; Jung and Chong, 2014). According to load theory (Lavie, 1995; Lavie et al., 2004), if the processing of the relevant information takes up all available resources, the processing of the irrelevant information would be reduced. In Experiment 1, remembering the digit stimuli took more resources under the high relative to low working memory load, and thus less resources were allocated to the processing of the irrelevant orientation information (i.e., the TAE task), resulting in the reduced magnitude of the TAE under the high-load condition. Our results provide first evidence that visual working memory load reduced the TAE.

Although the grating adaptors were not the attentional focus during adaptation stage in the present study, significant TAEs were still found in all experiments. This is in line with the results of previous studies which showed that TAEs still existed for the unattended gratings (Spivey and Spirn, 2000; Festman and Ahissar, 2004; Jung and Chong, 2014; Pavan et al., 2016). Even significant TAEs were found when the grating adaptors were rendered invisible (Jung and Chong, 2014). However, the magnitudes of the TAEs were reduced when the unattended or invisible grating adaptors were presented (Spivey and Spirn, 2000; Festman and Ahissar, 2004; Jung and Chong, 2014; Pavan et al., 2016), similar to the findings of Experiment 1. Therefore, these results indicate that adaption to orientation information (i.e., the grating adaptor) is, to some degree, an automatic processing but is modulated by high-level cognitive processes such as attention and working memory load. Our findings contribute to the notion that mutual interaction between the high-level and low-level processing in visual perception take places.

However, the results of Experiment 1 are inconsistent with previous studies regarding the effects of cross-modal load on the adaptation aftereffects. Zäske et al. (2016) investigated the effects of cross-modal working memory load on the adaptation aftereffects, and found that high visual working memory load increased the magnitude of the voice gender aftereffect, contrary to our current results. However, in Experiment 4 of Moradi et al. (2005), they found that cross-modal auditory load did not affect face identity aftereffects compared to the passive viewing condition. Similarly, the duration of motion aftereffect was also unaffected by the level of auditory load (Rees et al., 2001). Interestingly, the results of Experiment 3 in Moradi et al. (2005) showed a weaker face identity aftereffect under a highly attention-demanding visual load condition relative to a passive viewing condition, in line with our current results. Thus, these inconsistent results indicate that the effects of the cross-modal working memory load on the adaptation effects are different with those of the same modal. More work is needed to clarify the role of different processing modalities recruited by the working memory load toward different types of visual adaptation (e.g., whether an auditory working memory load could still reduce the magnitude of the TAE).

Future research would investigate whether our current findings could generalize to other types of visual adaptation regarding the role of working memory load on visual adaptation. Especially, contradictory results have been found regarding the role of attention and distraction on some types of visual adaptation. For example, some studies have shown that attention load failed to modulate the motion aftereffects (e.g., Morgan, 2011, 2012, 2013; Morgan and Solomon, 2019); however, other studies have showed that a high-attention load during adaptation decreased the strength of the motion aftereffects (e.g., Chaudhuri, 1990; Rees et al., 1997; Taya et al., 2009; Bartlett et al., 2018).

The finding of Experiment 1 failed to generalize to another type of working memory task in Experiment 2, which showed no significant difference on the magnitude of the TAEs between the high- and low-load condition. A potential factor for the null result was task difficulty in the working memory load task in Experiment 2. Whether for the high-load condition or the low-load condition, in Experiment 2 participants were required to remember two features of the stimuli (i.e., color and shape) for the working memory task. This requirement might render that more perceptual resources were also taken up even under the low-load condition. As a result, the working memory load effects were more difficult to detect in Experiment 2. Future research will examine whether the magnitude of TAEs would be reduced under the high relative to low load condition when only one feature (color or shape) was controlled in the working memory task.

In addition, for both experiments we unexpectedly found that there were no significant differences between the magnitudes of TAEs under the no-load condition and those under the high- or low-load condition. A limit regarding the design of the no-load condition was worth considering. There was a marked visual difference regarding the adaptor displays between the no-load condition and the high- or low-load condition. The adaptor gratings were presented with streams of digits or color-shape conjunctions placed at their center for the high- or low-load condition, whereas only adaptor gratings were presented for no-load condition. This visual difference produced an additional mismatch between these conditions. We had wanted to follow the passive viewing condition of previous studies in which only adaptors were presented generally (e.g., Moradi et al., 2005; Blaser and Shepard, 2009; Rhodes et al., 2011). A better-matched design is that random digits or color-shape conjunctions will be also presented at the center of the adaptor gratings in the no-load condition in future work.

Another limit of the current study was that we failed to disentangle memory load effect from attention load effect. It was possible that the high and low attentional load was included in high and low working memory load, respectively. Given that controlled attention and working memory maintenance have a shared cognitive resource (for a recent review, Oberauer, 2019), it is difficult to distinguish these two load effects when using the current paradigm. In fact, previous studies examining the role of working memory on visual aftereffects such as motion aftereffects (Blaser and Shepard, 2009) and face aftereffects (Moradi et al., 2005) also failed to disentangle memory load effect from attention load effect. Thus, attention load could be controlled when future work will examine how different levels of working memory load would modulate visual aftereffects.

In summary, by combining the working memory load task and the TAE task, we found that the high relative to low working memory load reduced the magnitude of the TAEs for the digit memory stimuli (Experiment 1) but not for the stimuli of the color-shape conjunctions (Experiment 2). These results provided mixed evidence regarding the modulation effects of the working memory load on the TAE, and further replications (e.g., using larger sample sizes for greater statistical power) are needed in future research.

## Data Availability Statement

The raw data of the current study are available at Open Science Framework: https://osf.io/grwsf/?view_only=3ed1ba0add1e4871b97499f5fb27183b.

## Ethics Statement

The studies involving human participants were reviewed and approved by the School of Psychology Ethics Committee at Guizhou Normal University. The patients/participants provided their written informed consent to participate in this study.

## Author Contributions

GM and YP designed the experiments. MC and XL collected the data. GM and MC performed data analysis. GM, MC, and SQ wrote the paper. All authors contributed to the article and approved the submitted version.

## Conflict of Interest

The authors declare that the research was conducted in the absence of any commercial or financial relationships that could be construed as a potential conflict of interest.
